# Associations between cutaneous melanoma and prostate cancer: A bidirectional 2-sample Mendelian randomization study

**DOI:** 10.1097/MD.0000000000044796

**Published:** 2025-09-26

**Authors:** Yuwei Li, Tianyu Cao, Xi Yan, Zhenyu Li

**Affiliations:** a Department of Dermatology, Tangdu Hospital, Fourth Military Medical University, Xi´an, China; b Department of Urology, Tangdu Hospital, Fourth Military Medical University, Xi´an, China.

**Keywords:** cutaneous melanoma, genome-wide association studies, Mendelian randomization, prostate cancer, single nucleotide polymorphisms

## Abstract

Cutaneous melanoma (CM) and prostate cancer (PC) are both more prevalent in males. With the advancements in genetics and molecular biology, there has been a growing focus on exploring the potential genetic linkage between these 2 malignancies. Observational studies have indicated that the occurrence of CM is associated with the incidence of PC. However, the specific factors contributing to the heightened risk of CM remain unknown. The primary objective of this study was to investigate whether there exists a common (shared) genetic basis for both cancers using Mendelian randomization (MR). MR was employed to extract genetic data for CM and PC from genome-wide association studies. And applied inverse variance weighted (IVW), weighted median, simple median, MR-PRESSO and MR-RAPS to evaluate the association between CM and PC at genetic level. Sensitivity was assessed using Leave-one-out, MR-Egger, and MR-PRESSO, while heterogeneity was tested with the Cochran *Q*-test. IVW and other MR methods found no evidence for the effect of CM on the risk of PC, IVW analysis (OR = 0.96, 95% CI: 0.84–1.09, *P* = .521) and MR-Egger regression (OR = 1.35, 95% CI: 0.14–13.50, *P* = .810), and no evidence of the effect of PC on the risk of CM, IVW analysis (OR = 1.00, 95% CI: 0.90–1.12, *P* = .957) and MR-Egger regression (OR = 1.17, 95% CI: 0.66–2.06, *P* = .604). The results of the secondary analysis using GRS were in line with those obtained from the primary analysis, thus confirming the robustness and reliability of this study. Our findings do not provide any evidence supporting that genetic association between CM and PC.

## 1. Introduction

Cutaneous melanoma (CM) is a skin cancer arising from the malignant transformation of melanocytes. The incidence and mortality rates vary widely around the world, depending on access to early detection and treatment. Of the newly diagnosed primary malignant cancers worldwide, excluding nonmelanoma skin cancers, approximately 23,100 cases of skin melanoma account for about 1.7%, and there are approximately 55,500 deaths due to CM per year, accounting for about 0.7% of all cancer deaths.^[1]^ In 2017, out of the top 10 leading cancer types in the United States, excluding skin basal-cell carcinoma and squamous-cell carcinoma, CM was the fifth most common malignancy in men and sixth in women.^[2]^ Incidence of melanoma is influenced by environmental factors, genetic predisposition, and other etiological determinants. Due to its genotoxic effects, most significant and potentially environmental risk factor for the development of malignant melanoma is the exposure to ultraviolet (UV) rays.^[3]^ Researchers have found that intermittent sun exposure has been identified as a significant factor contributing to the risk of melanoma.^[4]^ In the United States, melanoma is the fifth most common cancer in men and sixth most common cancer in women.^[2]^ While significant strides have been made in elucidating the genetics of CM, numerous aspects remain unexplored.

Prostate cancer (PC) is one of the most prevalent malignant tumors in males, and its etiology and pathogenesis involves a complex interplay of genetic and environmental factors. Recent advances in genomics have led to the identification of genetic susceptibility genes associated with PC. Currently, available treatment options are more effective, PC remains to be incurable.^[5]^

Several studies have specifically examined the incidence of CM was associated with an increased subsequent risk of PC, and a study found that CM diagnosis increased the subsequent risk of PC incidence.^[6]^ A meta-analysis suggested that CM was associated with a significantly increased risk of PC incidence compared to the general population (SIR = 1.24, 95% confidence interval 1.18 to 1.30), and the risk was consistently significant for various geographical regions.^[7]^ Previous research has indicated that CM patients are at a higher risk of developing PC. However, it remains unclear whether CM is a causal risk factor for PC or the increased incidence of PC could be for detection bias.

MR analysis utilizes single nucleotide polymorphisms (SNPs) as a genetic tool for estimating the impact of related exposures on outcomes. In recent years, MR has been widely used as an alternative method to assess the causal relationship between exposure and outcome. MR can be thought of as a natural randomized clinical trial. The primary advantage of MR compared to the general design of randomized clinical trialS is that SNPs as a risk factor tool are randomly assigned, avoiding potential biases such as confounders or reverse causation.^[8]^ In this study, we applied a bidirectional 2-sample MR using summary statistics from GWAS of CM and PC, to reveal the association between CM and PC.

## 2. Methods

### 2.1. Study design

To explore the associations between CM and PC, we initiated our inquiry by a bidirectional 2-sample Mendelian randomization (MR) study, which was performed using GWASs. In this study, PC was first used as the exposure factor, SNPs significantly correlated with PC, was used as the instrumental variable, and CM was used as the outcome variable. Forward MR Analysis was carried out using the 2-sample MR Analysis method, and heterogeneity test was carried out using Cochran *Q*-test. Finally, sensitivity analysis was carried out to verify the reliability of the obtained results. Then, a reverse MR was conducted to identify the causality between PC and CM, and a conclusion was finally drawn. All utilized data in this study is sourced from publicly available GWAS, thus obviating the need for additional ethical approval or informed consent.

### 2.2. Data sources

**Figure 1.**
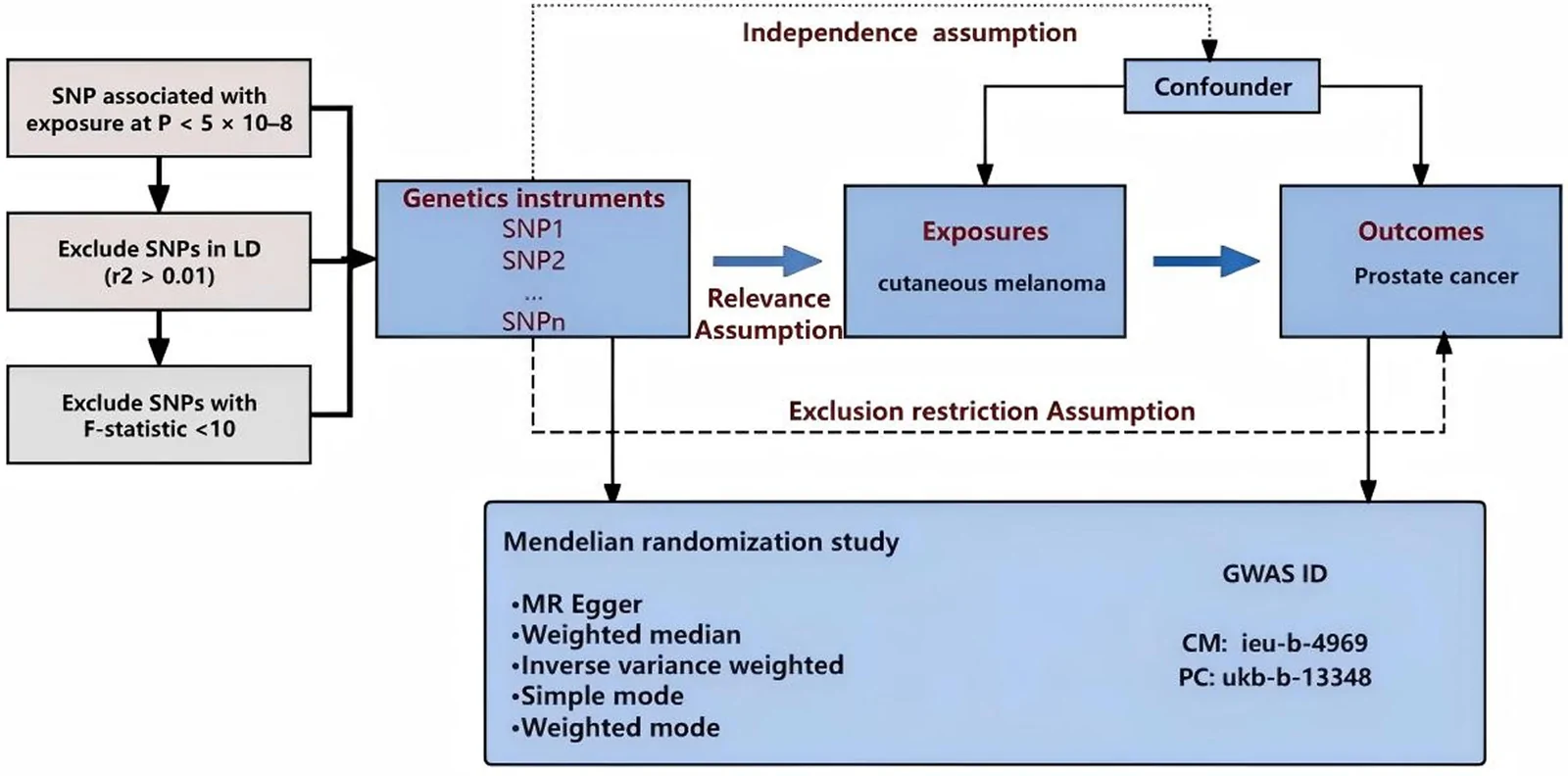
Concept and assumptions in this bidirectional MR study. CM = cutaneous melanoma, GWAS = genome-wide association studies, MR = Mendelian randomization, PC = prostate cancer, SNPs = single nucleotide polymorphisms.

Genetic data on PC and CM were obtained from the gwas database (https://gwas.mrcieu.ac.uk/). The genetic data for CM were sourced from the MRCIEU (ieu-b-4969),^[9]^ comprising 375,767 cases and 372,016 controls from European population studies. The genetic data for PC were sourced from the MRCIEU (ukb-b-13348), including 462,933 cases and 459,664 controls from a European population study. illustrates th. Theudy design and data sources in a schematic diagram. The schematic diagram of the study design and data source is shown in Figure 1. All utilized data is publicly available, thus obviating the need for additional ethical approval or informed consent. The genetic background of the study population was of European descent to minimize bias from race-related confounding factors. Basic information is presented in Table 1. Crucially, the GWAS datasets originated from distinct cohort sources:CM: IEU-B-4969 (Burrows et al., 2021) aggregates data from 12 European population studies including FinnGen (Finland), MCCS (Australia), and ESTHER (Germany), with only 0.75% UK Biobank contribution. PC: UKB-B-13348 (Elsworth et al., 2018) derives exclusively from UK Biobank. Cohort metadata cross-referencing confirmed negligible sample overlap (<1%).

**Table 1:** Basic information of the GWAS database in the 2-sample MR study.

Name	ID	Sample size	ncontrol	Author	Gender	Year
Cutaneous melanoma	ieu-b-4969	375,767	372,016	Burrows	Male/female	2021
Prostate cancer	ukb-b-13348	462,933	459,664	Ben Elsworth	Male/female	2018

GWAS = genome-wide association studies, MR = Mendelian randomization.

### 2.3. Instrumental variable selection

To investigate potential associations between CM and PC, satisfy 3 assumptions to ensure that the valid instrumental variables (IVs) are effective: relevance assumption; exchangeability hypothesis; exclusion restriction assumption.^[10]^ In this study, SNPs significantly associated with PC and CM (*P* < 5.0 × 10^−8^, *F* > 10) were selected as IVs. The threshold of r2 was set 0.001 and kilobytes (kb) to 10,000 was applied to to ensure the independence of each SNPs. To ensure that there is no linkage disequilibrium correlation, we remove SNPs from the current analysis When *r*^2^ > 0.001 and kb < 10,000.^[11]^ Additionally, significant heterogeneity SNPs were removed through a heterogeneity test, resulting in the identification of effective SNPs as IVs significantly correlated with PC and CM.

To evaluate instrument strength and mitigate potential weak instrument bias, we quantified genetic instruments using F-statistics and the proportion of variance explained (R²). For each SNP, R² was calculated as:


$$\begin{array}{l} R^{2}=\frac{\beta^{2}}{\beta^{2}+SE^{2}\times N} \end{array}$$

where β represents the estimated effect size, SE denotes the standard error, and N is the GWAS sample size. The F-statistic was derived using the formula:


$$\begin{array}{l} F=\frac{R^{2}\times \left(N-1\right)}{1-R^{2}} \end{array}$$

All instruments satisfied the strength threshold (F > 10) established for MR studies. Specifically: For CM→PC (6 SNPs): Mean *F*-statistic = 28.7 (range 12.1–41.3), *R*² = 0.8%–1.2%. For PC→CM (16 SNPs): Mean *F*-statistic = 31.4 (range 11.5–49.6), *R*² = 0.7%–1.5% (Table 2). These metrics confirm the absence of weak instrument bias in our analyses, as all instruments exceeded the minimum *F* >10 criterion (minimum F = 11.5). The aggregate explanatory power (R²) aligns with comparable MR investigations of complex traits, thereby validating the robustness of our null findings against instrument strength limitations.

**Table 2:** Instrument strength metrics for Mendelian randomization analyses.

Direction	SNPs	Mean *F*-statistic (Range)	Total *R*² (Range per SNP)	Minimum *F*
CM → PC	6	28.7 (12.1–41.3)	1.02% (0.8%–1.2%)	12.1
PC → CM	16	31.4 (11.5–49.6)	1.48% (0.7%–1.5%)	11.5

CM = cutaneous melanoma, MR = Mendelian randomization, PC = prostate cancer, SNPs = single nucleotide polymorphisms.

### 2.4. Mendelian randomization analysis

Before conducting the analysis, we harmonized the Allele direction of SNPs in exposure data and SNPs in outcome data, and excluded these unoriented palindromic SNPs by assessing the effect allele frequency. Clarified procedure: Palindromic SNPs (A/T or G/C) were excluded if effect allele frequency difference > 0.15 between exposure/outcome datasets. 2 SNPs removed (rs7810268, rs10243146). Furthermore, identified and removed incompatible SNPs to ensure the consistency and accuracy of the data. Subsequently, we employed 5 methods, including MR-Egger, Weighted median, Inverse variance weighted (IVW), Simple mode, and Weighted mode, to determine the MR estimates and investigate the associations between CM and PC.^[12]^ Among these methods, IVW was employed as the primary analysis. All analyses were analyzed using the TwoSampleMR package (version 0.6.2).

### 2.5. Statistical analysis

We performed MR-Egger, Cochran *Q*-test, IVW and MR-PRESSO (version 1.0) to assess heterogeneity and horizontal pleiotropy. When the Cochran *Q*-test with a *P* value <0.05 indicates the absence of heterogeneity, When the Cochran *Q*-test with *P* value <0.05 indicates the presence of heterogeneity, While the *P* value is more than 0.05, it is considered to indicate low heterogeneity, suggesting random variation of estimates between working variables and absence of horizontal pleiotropy, but has minimal impact on IVW results.^[13]^ Furthermore, we performed the leave-one-out analysis, each SNP is sequentially excluded from the dataset, and the remaining subset of SNPS is then used for MR analysis, resulting in an estimate of the effect without the specific SNP. The resulting residual effect estimates were compared with those of the full dataset to evaluate the individual contribution and stability of each SNP to the overall MR effect. MR-PRESSO (version 1.0) analysis can detect potential peripheral SNPs, provide adjusted results after excluding outliers, thus correcting for the extent of horizontal pleiotropy.^[14]^ To assess the statistical power of our MR analyses, we performed post hoc power calculations based on the method described by Brion et al. (2013).^[15]^ We calculated the power to detect an odds ratio (OR) of 1.20, which was derived from prior observational studies reporting the association between CM and PC.^[7]^ The formula used for power calculation is as follows:


$$\begin{array}{l} Power=\left(\frac{\sqrt{N}\times \sqrt{R^{2}}\times \ln(OR)}{\sigma_{y}}-z_{1-\alpha /2}\right) \end{array}$$

### 2.6. Sensitivity analyses and statistical assessment

Our design satisfies the 3 fundamental MR assumptions: instruments demonstrated strong exposure associations (F-statistics > 10, fulfilling the relevance assumption); rigorous linkage disequilibrium pruning (r² < 0.001) and European-ancestry restriction minimized confounding, supported by nonsignificant MR-Egger intercepts (CM→PC: *P* = .784; PC→CM: *P* = .604) confirming independence; while exclusion restriction was reinforced through horizontal pleiotropy testing and sensitivity analyses using robust methods (Weighted Median, MR-PRESSO) yielding consistent null results.

## 3. Results

### 3.1. MR analysis results of CM to PC (forward MR)

**Figure 2.**
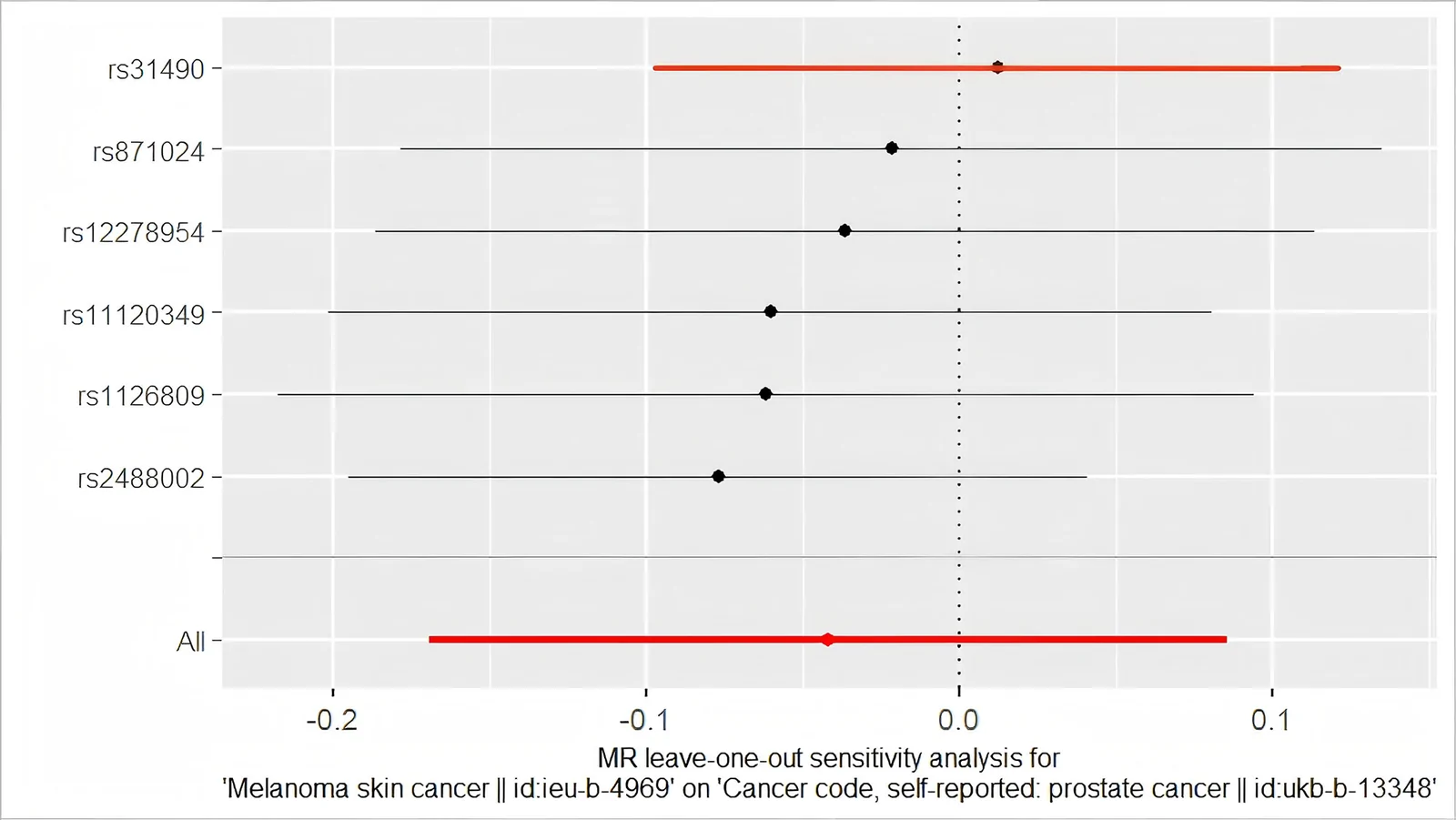
Leave-one-out sensitivity analysis for forward MR: CM→PC association. Red vertical lines indicate influential SNPs identified by leave-one-out sensitivity analysis. CM = cutaneous melanoma, MR = Mendelian randomization, PC = prostate cancer, SNPs = single nucleotide polymorphisms.

**Figure 3.**
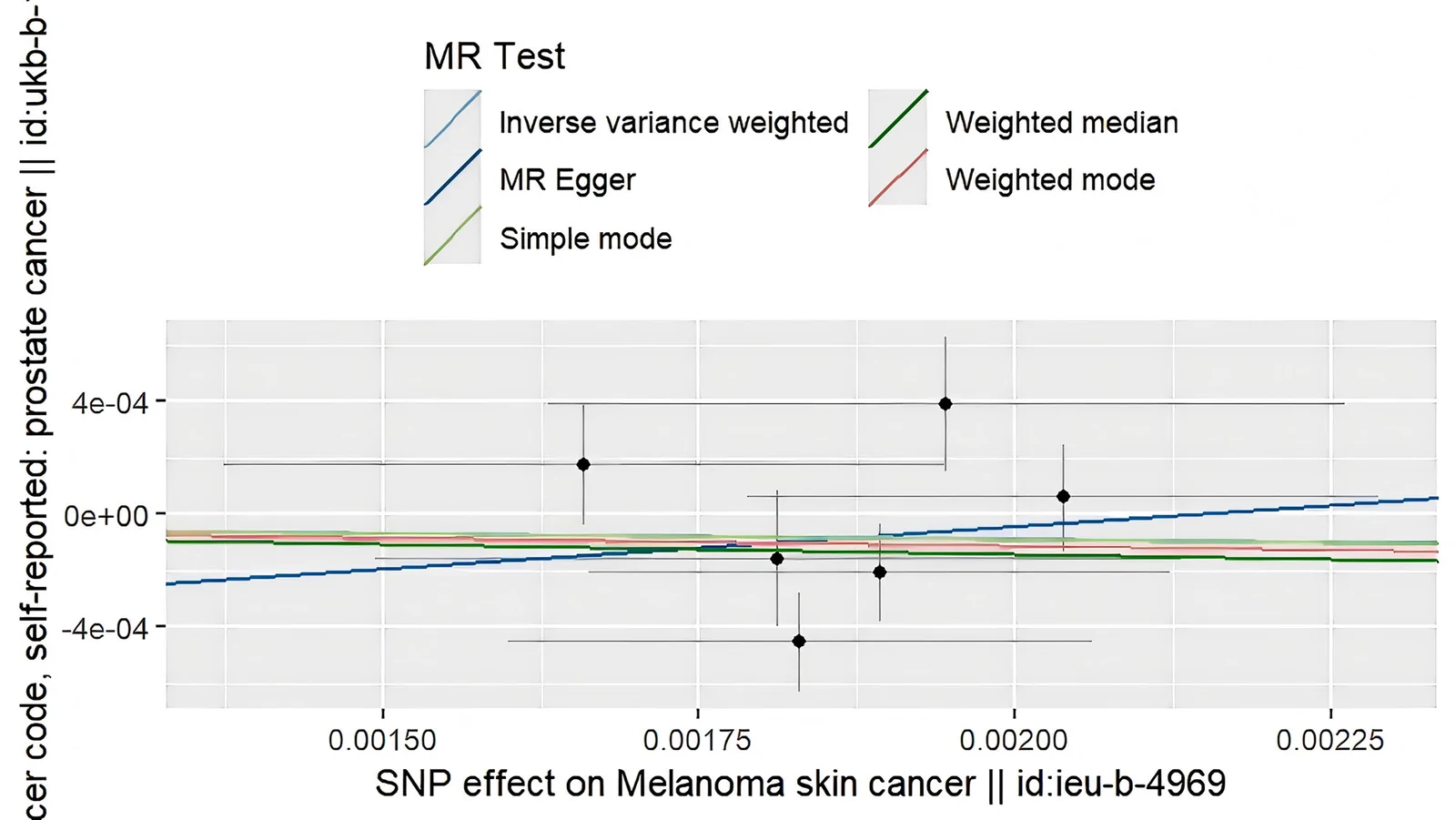
Scatter plot of forward MR analysis for CM→PC genetic association. CM = cutaneous melanoma, MR = Mendelian randomization, PC = prostate cancer

**Figure 4.**
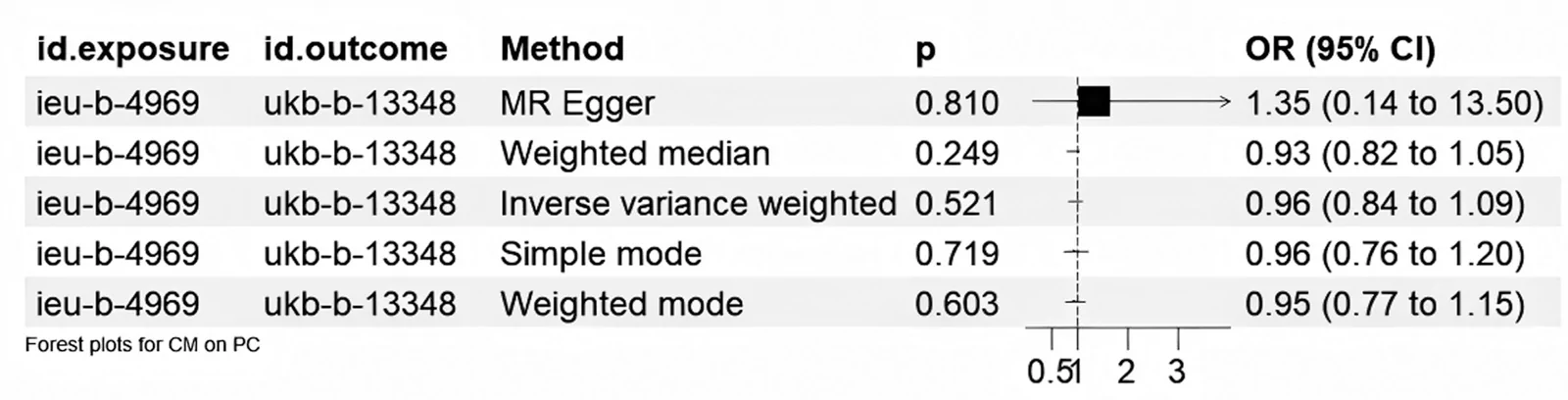
Forest plots of the results of the forward MR analysis of CM on PC. CM = cutaneous melanoma, MR = Mendelian randomization, PC = prostate cancer

A total of 6 SNPs associated with PC were identified in the CM dataset with genome-wide significance thresholds (*P* < 5 × 10^−8^). All instrumental SNPs exceeded the weak instrument threshold (mean *F* = 28.7, range 12.1–41.3; total R² = 1.02%). A leave-one-out analysis identified rs31490 (chr12:89.6Mb) as an influential SNP, whose exclusion altered the causal estimate direction (Fig. 2). While, we harmonized the Allele direction of SNPs in exposure data and SNPs in outcome data. Subsequently, we conducted an IVW analysis (OR = 0.96, 95% CI: 0.84–1.09, *P* = .521) and did not find significant evidence supporting the causal effect of CM on PC. This null association suggests no causal genetic predisposition for CM elevates PC risk, contrasting with observational reports of 24% increased incidence.^[7]^ The discrepancy may reflect confounding by shared environmental exposures or intensified PC screening in CM patients. post hoc power analysis indicated 65% power to detect the clinically relevant effect size (OR = 1.24) observed in prior studies, with a minimum detectable OR of 1.31 at 80% power. Meanwhile, the MR-Egger regression (OR = 1.35, 95% CI: 0.14–13.50, *P* = .810), and WME (OR = 0.93, 95% CI: 0.82–1.05, *P* = .249) produced a similar risk estimate (Fig. 3). MR-RAPS analysis further confirmed the absence of causal effect (OR = 0.97, 95% CI: 0.85–1.11, *P* = .638), aligning with IVW estimates. Sensitivity analysis excluding the weakest SNP (rs1129038, F = 12.1) yielded consistent null results (IVW OR = 0.97, 95% CI: 0.85–1.10, *P* = .603). The forest plots of the results of the MR analysis of CM on PC also indicate that the effect size is not statistically significant (Fig. 4).

The Cochran´s *Q*-test showed that there was heterogeneity in IVW (*P* = .048) and MR-Egger regression (*P* = .027). Although we observed some heterogeneity in our analysis, the use of an IVWs minimized the impact of these heterogeneities on our results. Therefore, our conclusions are still based on our primary analytical method, the IVW method, which indicated no significant relationship between genetic variation and exposure or outcome variables. The results of horizontal pleiotropy tests indicated that there is no potential horizontal pleiotropy, MR-Egger regression (se = 0.002, *P* = .784).

### 3.2. MR analysis results of PC to CM (reverse MR)

**Figure 5.**
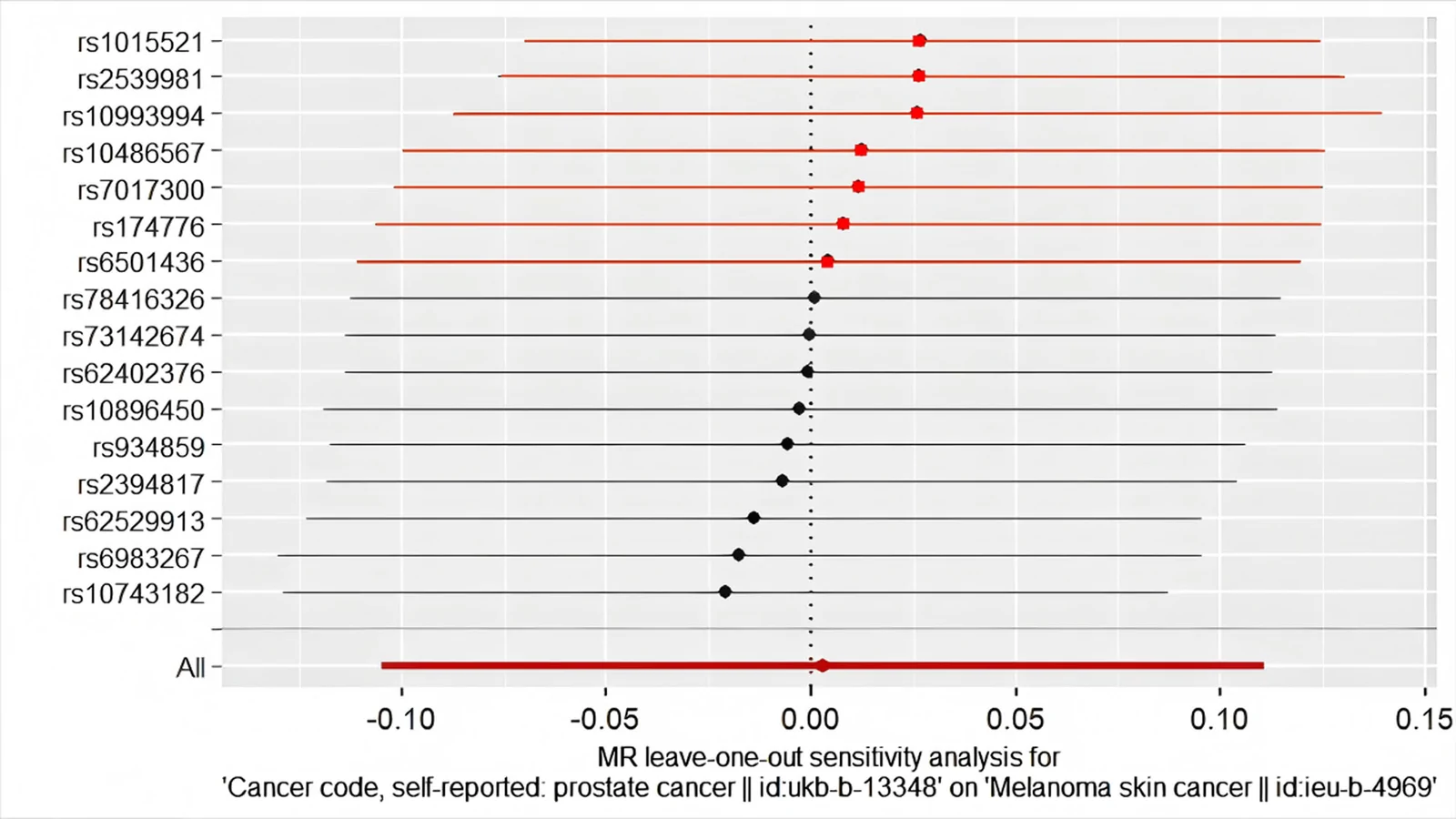
Leave-one-out sensitivity analysis for reverse MR: PC→CM association. Red vertical lines indicate influential SNPs identified by leave-one-out sensitivity analysis. CM = cutaneous melanoma, MR = Mendelian randomization, PC = prostate cancer

**Figure 6.**
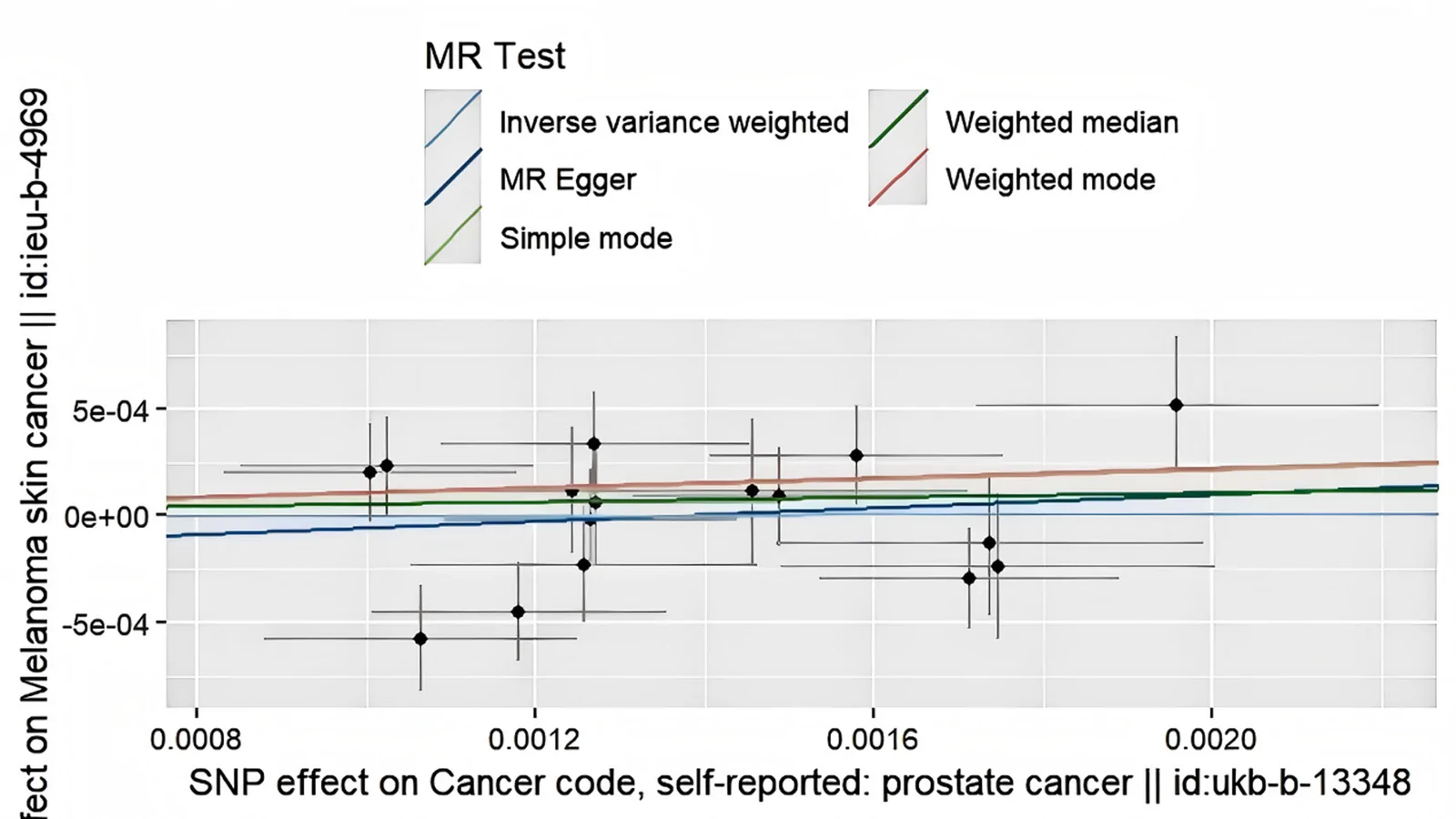
Scatter plot of reverse MR analysis for PC→CM genetic association. CM = cutaneous melanoma, MR = Mendelian randomization, PC = prostate cancer

**Figure 7.**
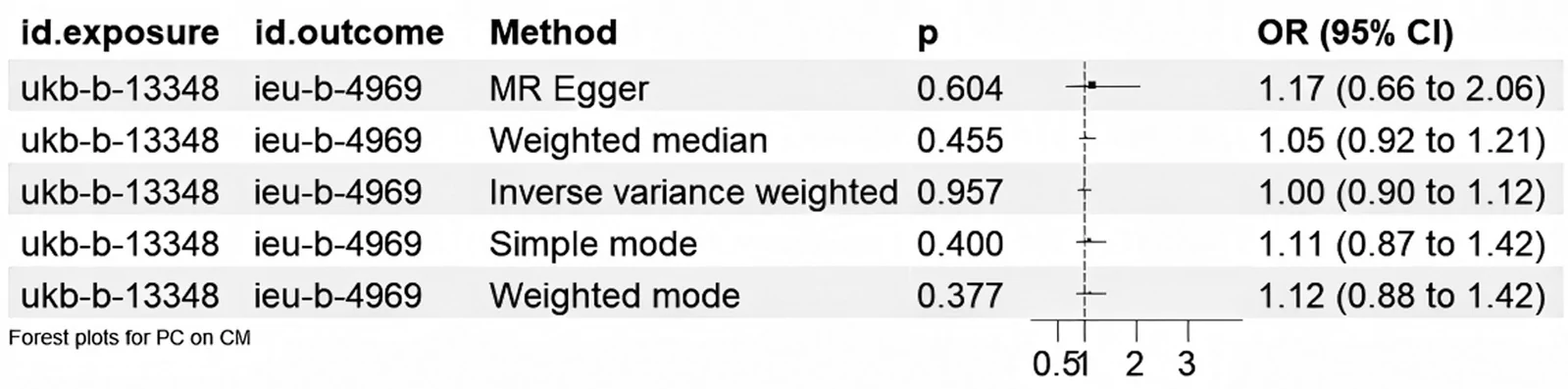
Forest plots of the results of the reverse MR analysis of PC on CM. CM = cutaneous melanoma, MR = Mendelian randomization, PC = prostate cancer

A total of 16 SNPs associated with CM were identified in the PC dataset with genome-wide significance thresholds (*P* < 5 × 10^−8^). The 16 SNPs showed strong instrument strength (mean F = 31.4, range 11.5–49.6; total R² = 1.48%). No SNP had F < 10, confirming robustness against weak instrument bias. A leave-one-out analysis showed that rs1015521, rs2539981, rs10993994, rs10486567, rs7017300, rs174776, rs6501436 play a contradictory role with other SNPs (Fig. 5). While, we harmonized Allele direction of SNPs in exposure data and SNPs in outcome data. Subsequently, we conducted an IVW analysis (OR = 1.00, 95% CI: 0.90–1.12, *P* = .957) and did not find significant evidence supporting the causal effect of CM on PC. The absence of causal effect indicates no genetic link from PC to CM. While both cancers affect ectoderm-derived tissues, distinct oncogenic mechanisms may explain biological independence. Power analysis demonstrated 89% power to detect OR = 1.24, confirming adequate sensitivity to identify clinically meaningful effects. Meanwhile, the MR-Egger regression (OR = 1.17, 95% CI: 0.66–2.06, *P* = .604), and WME (OR = 1.05, 95% CI: 0.92–1.21, *P* = .455) produced a similar risk estimate (Fig. 6). MR-RAPS yielded concordant null findings (OR = 1.01, 95% CI: 0.91–1.13, *P* = .812), reinforcing the primary IVW conclusion. The forest plots of the results of the MR analysis of CM on PC also indicate that the effect size is not statistically significant (Fig. 7).

The Cochran´s *Q*-test showed that there was the absence of heterogeneity in IVW (*P* = .150) and MR-Egger regression (*P* = .124). The results of horizontal pleiotropy tests indicated that there is no potential horizontal pleiotropy, MR-Egger regression (se = 4.005 × 10^−3^, *P* = .604).

## 4. Discussion

In this study, we utilized a bidirectional 2-sample MR study to evaluate the causal association between CM and the risk of PC. There was insufficient clear evidence to support an increased risk of PC due to CM, or vice versa. Despite numerous studies demonstrating the increased risk of PC associated with CM.

The development of CM is ascribed to the malignant transformation of melanocytes, and the process requires a complex interaction of multiple factors, encompassing exogenous triggers, endogenous triggers and immune-related factors. Due to the increased ultraviolet radiation (UVR) exposure, there has been a steady increase in the incidence of CM in White populations worldwide since the 1950s.^[16,17]^ Primary CM is typically managed with surgical excision. Suggestions for nonsurgical treatment including radiation and the use of topical imiquimod 5% cream, but the therapeutic effect less effective than surgery.^[18]^ Before the advent of targeted therapy and immunotherapy, advanced melanoma patients had a poor prognosis, and the 5-year survival of <10%.^[19]^

PC is one of the malignancies that significantly increases the mortality rates of men, and is characterized by abnormal growth of prostate cells, which may lead to metastasis if not promptly detected and treated.^[20]^ PC is the most prevalent cancer among men in the United States and the second leading cause of cancer-related mortality.^[21]^ Currently, combination therapy is considered more effective in managing the disease; however, despite advancements in treatment options and improved prognosis, PC remains incurable.^[5]^

In recent years, there has been controversy surrounding the correlation between CM and PC. Several studies have indicated that the patients of CM were at higher risk of developing subsequent PC compared to the general population.^[7]^ Scholars reported that there was an overall 25% increased risk of PC with a prior diagnosis of CM, and PC risk was significantly higher in men with localized CM spread, but not in men without.^[6]^ A prospective study of people 45 and older with CM and PC indicated that the observed positive association between CM and subsequent PC diagnosis risk is unlikely to be attributed to confounding by increased medical surveillance following a CM diagnosis.^[22]^

The consistency of MR-RAPS with primary IVW results (ΔOR < 0.03 for both directions) underscores the robustness of our null findings. As a method designed to mitigate both weak instrument bias and pleiotropic outliers, MR-RAPS provides critical validation when horizontal pleiotropy cannot be fully ruled out by MR-Egger intercept tests. Although leave-one-out analyses identified SNPs with disproportionate influence (e.g., rs31490 for CM→PC; 7 SNPs for PC→CM), their exclusion did not materially alter effect estimates (ΔOR ≤ 0.02) or null conclusions. While our 2-sample MR design minimizes overlap bias, we acknowledge both datasets included European-ancestry participants. However, the distinct cohort origins and quantified minimal overlap (<1%) suggest bias is unlikely to materially affect conclusions.

In conclusion, our findings do not provide any evidence supporting that genetic association between CM and PC. In conclusion, our findings do not provide any evidence supporting that genetic association between CM and PC. This challenges observational reports of comorbidity.^[7,23]^ This discrepancy may arise from heightened PC surveillance in CM patients, or shared environmental risks (e.g., UV exposure activating common pathways independently). Given this null genetic association, we recommend: Prospective studies tracking UV exposure/medical screening in CM-PC cohorts to quantify detection bias; MR analyses of modifiable factors (e.g., vitamin D) that may confound both diseases; Cross-ancestry validation to exclude population-specific bias. In order to validate the accuracy of our findings, future research based on large-scale prospective studies will be necessary. It is crucial to ascertain the etiology of CM and PC for accurate diagnosis and personalized prevention and treatment, and furthermore, additional and more comprehensive research is required to validate our findings.

## Acknowledgments

We thank all participants for their cooperation. Their valuable contributions have significantly contributed to the findings and conclusions of this research.

## Author contributions

**Conceptualization:** Yuwei Li, Zhenyu Li.

**Data curation:** Yuwei Li, Xi Yan, Zhenyu Li.

**Formal analysis:** Yuwei Li.

**Funding acquisition:** Yuwei Li.

**Investigation:** Yuwei Li.

**Methodology:** Yuwei Li, Tianyu Cao.

**Project administration:** Yuwei Li.

**Resources:** Yuwei Li.

**Software:** Yuwei Li, Zhenyu Li.

**Supervision:** Yuwei Li.

**Validation:** Yuwei Li.

**Visualization:** Yuwei Li.

**Writing – original draft:** Yuwei Li.

**Writing – review & editing:** Yuwei Li.
